# INCIDENCE AND ASSOCIATED FACTORS OF DEMENTIA IN PEOPLE WITH SCHIZOPHRENIA: A POPULATION-BASED COHORT STUDY

**DOI:** 10.1093/geroni/igac059.1815

**Published:** 2022-12-20

**Authors:** Yingyang Zhang, Pengcheng Wang, Jennifer Y M Tang, Gloria H Y Wong, Hao Luo

**Affiliations:** The University of Hong Kong, Hong Kong, Hong Kong; The University of Hong Kong, Hong Kong, Hong Kong; The University of Hong Kong, Hong Kong, Hong Kong; The University of Hong Kong, Hong Kong, Hong Kong; The University of Hong Kong, Hong Kong, Hong Kong

## Abstract

Existing evidence suggests that people with schizophrenia may have an increased risk of dementia. This study aimed to quantify the incidence of dementia and examine putative risk factors associated with dementia in people with schizophrenia. This is a cohort study using population-based electronic health records of people who visited public hospitals in Hong Kong. We included participants (≥ 45 years) with schizophrenia diagnosis between 2009 to 2018 without dementia and followed them until March 2021. Incidence of dementia was calculated, and Cox proportional hazard regression was utilized to estimate hazard ratios of dementia, adjusting for covariates. A total of 20,901 individuals (mean [SD] age, 58.4 [10.3] years, 55.7% were women) were followed for 154,630 person-years (median [interquartile range] follow-up, 7.6 [4.7-10.3] years). The incidence of dementia was 5.1 [95% CI, 4.7-5.4] per 1000 person-years (those aged 45-64: 2.0 [1.8-2.3]; those 65 and above: 17.0 [15.6-18.5] per 1000 person-years). Factors independently associated with all-cause dementia were age (Hazard Ratio, 1.11 [95% CI, 1.10-1.11]), diabetes (1.62 [1.36-1.93]), bipolar disorders with at least five-year duration (2.12 [1.50-3.01]), and schizophrenia duration (1.02 [1.01-1.04]). Stratified analysis indicated that the association of these factors with dementia had differences in different sex and age groups. The incidence of dementia after schizophrenia diagnosis was high and two risk factors of dementia that are different from the well-established ones were identified.

